# Breast Cancer Resistance Protein (BCRP/*ABCG2*) Inhibits Extra Villous Trophoblast Migration: The Impact of Bacterial and Viral Infection

**DOI:** 10.3390/cells8101150

**Published:** 2019-09-26

**Authors:** Phetcharawan Lye, Enrrico Bloise, Lubna Nadeem, Chun Peng, William Gibb, Tania M. Ortiga-Carvalho, Stephen J. Lye, Stephen G. Matthews

**Affiliations:** 1 Department of Physiology, University of Toronto, Toronto, ON M5S 1A8, Canada; tlye@lunenfeld.ca (P.L.); lye@lunenfeld.ca (S.J.L.); Stephen.Matthews@utoronto.ca (S.G.M.); 2 Department of Morphology, Federal University of Minas Gerais, Belo Horizonte 31270-901, Brazil; 3 Lunenfeld-Tanenbaum Research Institute, Mount Sinai Hospital, Toronto, ON M5G 1X5, Canada; nadeem@lunenfeld.ca; 4 Department of Biology, York University, Toronto, ON M3J 1P3, Canada; cpeng@yorku.ca; 5 Department of Obstetrics & Gynaecology, University of Ottawa, Ottawa, ON K1H 8L6, Canada; william.gibb@uottawa.ca; 6 Department of Cellular & Molecular Medicine, University of Ottawa, Ottawa, ON K1H 8M5, Canada; 7 Laboratory of Translational Endocrinology, Biophysics Institute Carlos Chagas Filho, Federal University of Rio de Janeiro, Rio de Janeiro 21941-902, Brazil; taniaort@biof.ufrj.br; 8 Department of Obstetrics & Gynaecology, University of Toronto, Toronto, ON M5G 1E2, Canada; 9 Department of Medicine, Faculty of Medicine, University of Toronto, Toronto, ON M5S 1A8, Canada

**Keywords:** Breast cancer resistance protein (BCRP/*ABCG2*), extra villous trophoblast, first trimester placenta, migration, infection

## Abstract

Extravillous trophoblasts (EVT) migration into the decidua is critical for establishing placental perfusion and when dysregulated, may lead to pre-eclampsia (PE) and intrauterine growth restriction (IUGR). The breast cancer resistance protein (BCRP; encoded by *ABCG2*) regulates the fusion of cytotrophoblasts into syncytiotrophoblasts and protects the fetus from maternally derived xenobiotics. Information about BCRP function in EVTs is limited, however placental exposure to bacterial/viral infection leads to BCRP downregulation in syncitiotrophoblasts. We hypothesized that BCRP is involved in the regulation of EVT function and is modulated by infection/inflammation. We report that besides syncitiotrophoblasts and cytotrophoblasts, BCRP is also expressed in EVTs. BCRP inhibits EVT cell migration in HTR8/SVneo (human EVT-like) cells and in human EVT explant cultures, while not affecting cell proliferation. We have also shown that bacterial—lipopolysaccharide (LPS)—and viral antigens—single stranded RNA (ssRNA)—have a profound effect in downregulating *ABCG2* and BCRP levels, whilst simultaneously increasing the migration potential of EVT-like cells. Our study reports a novel function of BCRP in early placentation and suggests that exposure of EVTs to maternal infection/inflammation could disrupt their migration potential via the downregulation of BCRP. This could negatively influence placental development/function, contribute to existing obstetric pathologies, and negatively impact pregnancy outcomes and maternal/neonatal health.

## 1. Introduction

The placenta supports the growth and development of the fetus and is paramount to a successful pregnancy [1]. A critical stage of early placental development is the proliferation and differentiation of cytotrophoblasts (CT) into extravillous trophoblasts (EVTs). CTs originating at the tips of the chorionic villi first grow and establish cell columns before differentiation. The resulting EVTs invade the maternal uterine tissues either through the decidualized endometrium into the inner myometrium (interstitial) or through the lumen of the uterine spiral arteries against the blood flow (endovascular) [2,3,4].

While appropriate migration of EVT is essential for a successful pregnancy, the maternal decidua maintains tight control over EVT migration through paracrine signaling. Insufficient invasion can result in poor placentation and can lead to preeclampsia (PE), intrauterine growth restriction (IUGR), and other serious obstetric complications [5]. Conversely, uncontrolled EVT migration/invasion can lead to the deeper penetration of trophoblasts into the myometrium rather than decidua basalis (placenta acreta) [6] or even through the uterus, affecting the surrounding organs (placenta percreta) [7], resulting in maternal/fetal morbidity and mortality. Further, in certain circumstances, uncontrolled trophoblast proliferation and invasion can lead to choriocarcinoma [8].

The transmembrane efflux transporter, breast cancer resistance protein (BCRP, encoded by *ABCG2* gene), belongs to the ATP-binding cassette (ABC) transporter superfamily and is primarily localized to the apical brush-border membrane of the syncytiotrophoblast (ST) barrier [9]. BCRP regulates CT fusion into ST and provides embryo/fetal protection from drugs and environmental toxins that are present in the maternal circulation. In STs, BCRP prevents the transfer of a number of drugs from the maternal to the fetal circulation and is, therefore, considered an important fetal “gatekeeper” throughout pregnancy [9].

We have previously reported that human placental BCRP expression increases with advancing gestation and peaks at term [10,11]. Importantly, viral and bacterial challenges or pathological inflammatory states alter placental BCRP expression differently. Lipopolysaccharide (LPS; modelling bacterial infection) decreased *ABCG2* and BCRP expression in first trimester human placental explants (but not in third trimester explants). Whereas, polyinosinic:polycytidylic acid (poly(I:C) (a double-stranded viral antigen) did not induce changes in BCRP expression [12]. In sharp contrast, the placenta from preterm pregnancies complicated by chorioamnionitis exhibited increased *ABCG2* and BCRP expression [13]. This indicates that the nature (source) and timing (gestational age) of infection/inflammation determines the positive or negative effects on the regulation of BCRP expression and consequently the potential fetal exposure to harmful BCRP substrates.

*ABCG2* and BCRP expression are elevated in stem cells and cancer cells [14,15,16,17]. While BCRP is a membrane efflux protein, its role in regulating cancer cell function (cell proliferation, migration/invasion) has also been established. Studies have shown that BCRP induces cancer cell proliferation [14,18] and migration/invasion.

Together, these data suggest that infection and inflammation can modulate the expression of *ABCG2* and BCRP in placental trophoblasts. During early gestation, altered levels of BCRP may affect the migration and invasion potential of these cells, thereby causing pregnancy complications, though to date, no studies have tested this hypothesis. Given the relatively high incidence of bacterial and viral infections during early human pregnancy [19] and its impact on BCRP expression, we determined the role of *ABCG2* and BCRP in modulating the migration potential of EVTs, which is critical for the establishment of placentation in early pregnancy. Further, we determined the impact of bacterial (mimicked by LPS) or viral (mimicked by single stranded RNA, ssRNA) infection on these processes.

## 2. Materials and Methods

### 2.1. Ethical Approval

Healthy first trimester human placental tissue was collected at 7–10 weeks of pregnancy by the Research Centre for Women’s and Infants’ Health Bio Bank program at Sinai Health System after written informed consent (process n# 26573) and in adherence with the policies of the Sinai Health System and the University of Toronto Research Ethics Board.

### 2.2. Human Placental Explant Culture

First trimester human placentae (6 to 7 weeks) from the elective termination of singleton pregnancies were used to set up the extravillous explant culture as described earlier [20]. Briefly, small clusters of 2 to 3 column cytotrophoblasts (CCT) villi presenting high vascularization and clear white tips were excised under the dissecting microscope. Tips of the villi were cleared to expose CCT stem cells, which were gently spread on the matrigel (200 µL per insert of phenol red free, Becton Dickinson, Bedford, MA, USA) coated transwell inserts (Millipore Corp., Billerica, MA, USA) in a 24-well culture plate. Serum free culture medium (400 µL of DMEM/F12) supplemented with Normacin (1%, Invivogen, San Diego, CA, USA) was added to the wells beneath the inserts to keep the matrigel moist, and explants were allowed to adhere to the Matrigel overnight (37 °C, 3% O_2_, and 5% CO_2_) as described earlier [21]. The next day, 200 µL of medium was added to the inserts and the explants were incubated (for 24 h) to allow the formation of EVT outgrowths. Explant outgrowth was observed under a microscope. Only explants exhibiting EVT sprouting were included in the study. For *ABCG2* knockdown, explant media was supplemented with the si*ABCG2* transfection complexes or scrambled control (50 nmol/L—please see below). Explants were then photographed (at time zero: T0) using a Leica DFC400 camera (Leica Microsystems GmbH, Wetzlar, Germany) attached to a dissecting microscope. Photographs were taken after 24 h (T24) and 48 h (T48) post transfection. The area of outgrowth at T0, T24, and T48 was analyzed from the pictographs using imageJ software and percent growth was calculated by dividing the difference between the final (T24 or T48) and initial (T0) area of outgrowth with the final area of outgrowth *100. Each experiment was performed in triplicate with a total of N = 3 first trimester human placentae.

Explant tissues attached to Matrigel were collected by cutting the membrane from the transwell inserts, were washed once with PBS (1x), and were fixed with paraformaldehyde (4%, 1 h). The tissues were dehydrated in ascending grades of ethanol, cleared in xylene, and embedded in paraffin. Sections (5 μm) were mounted on slides for Immunohistochemistry.

### 2.3. Immunohistochemistry

Explants were processed for immunohistochemical analysis as previously described [10,22]. Briefly, slides were deparaffinized, rehydrated, and subjected to antigen retrieval with sodium citrate. After blocking with Dako protein block (Dako, Mississauga, ON, Canada), the slides were incubated overnight at 4 °C with primary antibodies: anti-mouse BCRP (1:200, BXP-21, ab3380, Millipore, Billerica, MA, USA), anti-mouse HLA-G (1:300, 4H84, Exbio, Burlington, ON, Canada), and anti-mouse CK7 (1:1000; M7018, Dako). In the controls, mouse or rabbit IgG1 (Dako) was added instead of the primary antibody. After incubation, the slides were washed and incubated with the corresponding biotinylated secondary antibody (1:300, 1 h, Dako). Sections were washed in 1× PBS (3 × 10 min) and incubated with streptavidin-HRP (30 min; Dako); immunostaining was detected with the peroxidase substrate kit DAB (Dako). The slides were counterstained with hematoxylin, dehydrated in ascending concentrations of ethanol, and cover slipped with mounting medium. Visualization was undertaken with an Olympus BX61 upright, motorized microscope, and representative images were captured using an Olympus DP72 digital camera (Olympus, Tokyo, Japan).

### 2.4. Cell Line and Culture

The first trimester human placental cell line HTR8/SVneo was kindly provided by Dr. Charles Graham, Queen’s University. The cell line was developed using first trimester EVTs infected with simian virus 40 large T antigen (SV40) [23]. HTR-8/SVneo cell culture was performed as previously described [20]. Briefly, cells were cultured in Roswell Park Memorial Institute 1640 medium (RPMI; Thermo Fisher Scientific, Wilmington, NC, USA) supplemented with 10% fetal bovine serum (FBS; Wisent, St Bruno, QC, Canada), 100 IU/mL of penicillin, and 100 IU/mL of streptomycin at 20% O_2_ (5% CO_2_, 37 °C) (Invitrogen Canada Inc., Burlington, ON, Canada). Cells were seeded (380,000 per well, respectively) in 6-well plates and cultured for 24 h at 20% O_2_ (5% CO_2_, 37 °C) and were then treated with LPS (0.1 or 1 µg/mL; Sigma-Aldrich, St. Louis, MO, USA) or ssRNA (1 or 2.5 µg/mL; Invivogen, San Diego, CA, USA) for 12 h in dose ranges previously shown to elicit a trophoblast inflammatory response [12,24,25]. Cells and media were then collected and stored at 80 °C for total RNA and protein extraction or enzyme-linked immunosorbent assay (ELISA).

### 2.5. Quantitative Real Time PCR (qPCR)

Total RNA was isolated from HTR-8/SVneo cell lines using RNeasy Plus Universal Mini Kit (Qiagen, Toronto, ON, Canada), as previously described [12,13,26]. RNA concentration and purity were assessed with a NanoDrop1000 Spectrophotometer (Thermo Scientific, Wilmington, NC, USA) and Experion RNA StdSens Analysis Kit (Bio-Rad, Mississauga, ON, Canada), respectively. RNA was reverse-transcribed into cDNA using the iScript Reverse Transcription Supermix (Bio-Rad). *ABCG2*, *IL-6*, *IL-8*, *CCL2*, *TRL-4*, and *TRL-7* mRNA levels were measured by qPCR using SYBR Green reagent (Sigma-Aldrich) and the CFX 380 Real-Time system C1000 TM Thermal Cycler (Bio-Rad), with the following cycling conditions: initial denaturation at 95 °C (2 min) followed by 39 cycles of denaturation at 95 °C (5 s), and combined annealing and extension at 60 °C (20 s). Gene expression was normalized to the geometric mean of selected reference genes including TATA-binding protein (*TBP*) and hypoxanthine-guanine phosphoribosyltransferase (*HPRT*), which had stable expression after LPS or ssRNA treatments in the HTR-8/SVneo cell line. The relative expression of target genes was calculated by the 2^−ΔΔCT^ method [27]. The primer sequences of all the assessed genes are shown in Table 1 [28].

### 2.6. Immunoblotting

Western blot analysis was conducted as previously described [11,12,13,22,26]. Briefly, protein isolated from cultured cells was extracted by sonication using lysis buffer (1 mol/L Tris-HCL pH 6.8, 2% SDS, 10% glycerol with added protease and phosphatase inhibitor cocktail; Thermo Scientific). The protein concentration was determined with the Pierce BCA Protein Assay kit (Thermo Scientific). Proteins were separated by electrophoresis (30 µg 100 V, 1 h) using SDS polyacrylamide gels (8%). Proteins were then transferred (10 min) to polyvinylidene fluoride (PVDF) membrane using Trans-Blot Turbo (Bio-Rad). Membranes were blocked with skim milk (5%; 1 h at room temperature). The primary antibodies used were anti-rabbit BCRP (dilution 1:3000; Abcam, ab63907, Toronto, ON, Canada) and anti-rabbit ERK2 (dilution 1:3000; Santa Cruz Biotechnology, Dallas, TX, USA). Blots were incubated with primary antibodies overnight at 4 °C. The PVDF membranes were subsequently incubated for 1 h with HRP-linked anti-rabbit secondary antibody (GE Healthcare Bio-Science, Baie d’Urfe, QC, Canada) at concentrations of 1:10,000 (ERK2) and 1:15,000 (BCRP). Protein-antibody complexes were detected by incubating (for 5 min) the PVDF membranes with Laminate Crescendo Western HRP Substrate (Millipore, Oak Drive, CA, USA) and chemiluminescence was detected under UV by using the ChemiDoc^TM^ MP Imaging system (Bio-Rad). The protein band intensity was quantified using Image Lab^TM^ software.

### 2.7. Enzyme-Linked Immunosorbent Assay (ELISA)

IL-6 and IL-8 concentrations in HTR-8/Vneo supernatant exposed to LPS, ssRNA, or VEH for 12 h were determined using a human IL-6/8 ELISA Development Kit (Invitrogen by Thermo Scientific, Wilmington, NC, USA) according to the manufacturer’s instructions (minimum detection limit, 2 pg/mL; intra-assay variation, 4.94%).

### 2.8. Transient Transfection with siRNA Oligonucleotides

Three different Stealth *ABCG2* siRNA duplexes, designed and synthesized by Shanghai GenePharma (Shanghai, China), were used for the transient inhibition of endogenous *ABCG2* in HTR8/SVneo cells. Sequences are provided in Table 2. HTR8/SVneo cells were seeded at a density of 13,000 cells/well in 96-well imagelock microplates (BioScience Inc., Ann Arbor, MI, USA). The following day, the cells were transfected using Lipofectamine RNAiMAX transfection reagent (2 µL/mL) according to the manufacturer’s protocol. All *ABCG2* siRNA duplexes were pooled in a cocktail (50 nM) in equal proportions. An equivalent amount of scrambled siRNA was used as a control. The cells were harvested 24 h post transfection for RNA analysis and 48 h for protein or migration analysis. 

### 2.9. Cell Migration Assay

To determine the effect of BCRP on HTR8/SVneo cell migration, 13,000 cells were seeded in 96-well imagelock plates. The next day, the cells were transfected with the 50nM pooled *ABCG2*-siRNAs (si*ABCG2*-1 + si*ABCG2*-2 + si*ABCG2*-3) or scrambled control (NC) and were incubated for 48 h for effective knockdown on endogenous *ABCG2*. Wounds were created across the diameter of each well using an automatic wound maker (Essen Biosciences, Ann Arbor, MI, USA) and the cells were washed twice (1× PBS) to remove the floating cells. The cells were then replenished with the serum-reduced medium (Opti-MEM) and incubated in the IncuCyte^®^ S3 system (Essen Bioscience, Ann Arbor, MI, USA), where all the wounds were imaged every 2 h for 36 h. Cell migration was determined as the percent wound density by the IncuCyte™ software from the data collected every 2 h through the 36 h. Percentage of Relative Wound Density (RWD) was determined by the Incucyte software while area under the curve (AUC) was calculated for each condition from mean RWD values to compare the treatments, as previously described [15].

The effect of pharmacological inhibition of BCRP on cell migration was assessed using the specific inhibitor Ko143 (Sigma-Aldrich). Cells were seeded at a density of 22,000 cells per well in imagelock plates and treated with Ko143 (1 µM) or its diluent (0.001% ethanol) as the vehicle for 2 h before they were wounded. The cells were wounded, washed, and imaged as described above. Ko143 (1 µM) or its vehicle was maintained in the medium (serum reduced) during the migration assay.

The effect of the infection challenges on HTR8/SVneo cell migration was assessed by seeding 22,000 cells per well in 96-well imagelock culture plates (Essen Biosciences, Ann Arbor, MI, USA). The following day, the cells were treated with LPS (0.1 or 1 µg/mL) or its vehicle (water) for 24 h. Similarly, cells were pre-treated with ssRNA (1 or 2.5 µg/mL) or its vehicle (water) for 24 h. The treatments were replenished after the wounded cells were washed and then they were imaged and analyzed by the IncuCyte^®^ S3 system over the next 36 h.

### 2.10. Cell Proliferation Assay

To verify if the effect of BCRP on cell migration is due to altered cell migration and not due to a secondary effect on cell proliferation, we replicated the conditions of the migration assay in regular 96-well culture plates and subjected them to cell proliferation assessment using the incuCyte system. Briefly, 5000 cells were seeded in 96-well culture plates. The next day, cells were transfected with the 50 nM pooled *ABCG2*-siRNAs, as described in the previous section, for 24 h and were then incubated in the IncuCyte^®^ S3 system (Essen Bioscience, Ann Arbor, MI, USA) in serum-reduced medium (Opti-Mem, similar to the migration assay), where cells were imaged every 2 h for the next 60 h. Cell proliferation was determined as the percent confluence by the IncuCyte™ software from the data gained every 2 h through the 60 h. Area under the curve (AUC) was calculated for each condition from mean % confluence values to compare the treatments as described previously [15]. The validity of the effect of pharmacological inhibition of BCRP and the effect of bacterial and viral infective challenges on cell migration (and not on cell proliferation) was similarly assessed, using approaches described above.

To determine the effect of BCRP on cell proliferation under the regular growth conditions, we transfected/treated the HTR8/SVneo cells as described in the previous section for 24 h and then incubated the cells in complete medium (with 10% FBS) and assessed cell proliferation through the incuCyte system as described above.

### 2.11. Statistical Analysis

All analyses were conducted blind to the experimental conditions. Data analyses were performed with Prism version 6 (GraphPad Software Inc., San Diego, CA, USA) and data were assessed for normal distribution using D’Agostino and Pearson or the Shiparo-Wilk test. Differences in migration and proliferation following the pharmacological and molecular inhibition of BCRP expression were assessed by unpaired *t*-test. Differences between the treatments were determined by one-way ANOVA followed by the Newman-Keuls post-hoc test.

## 3. Results

### 3.1. Expression of BCRP Is Enriched in Column Cytotrophoblasts (CCT) and Extravillous Trophoblasts (EVTs) in Placental Explants

**Figure 1:**
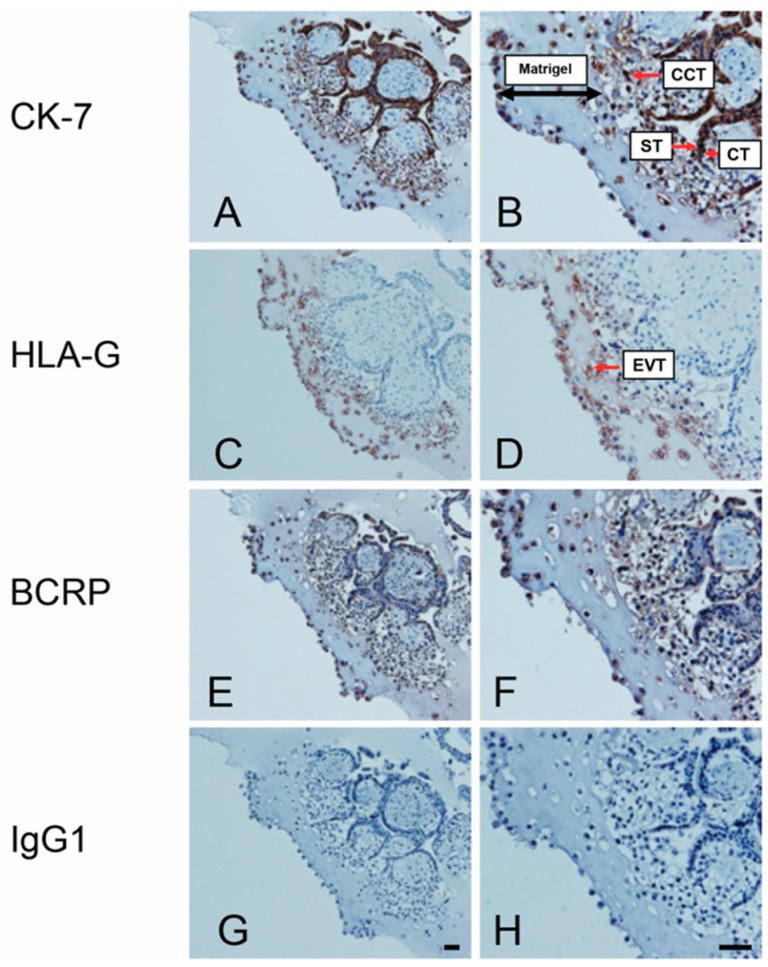
Breast cancer resistance protein (BCRP) is expressed in diverse trophoblast lineages including extravillous trophoblasts (EVT). Representative immunohistochemistry (IHC) images from tissue sections of first trimester human placental explants cultured on Matrigel (n = 6), showing: (**A**,**B**) cytokeratin 7 (CK7) expression (a trophoblast marker) in cytotrophoblast (CT), column cytotrophoblast (CCT), syncytiotrophoblast (ST), and extravillous trophoblast (EVT); (**C**,**D**) HLA-G expression in EVTs (EVT cell marker); (**E**,**F**) BCRP protein localization to CT, CCT, ST, and EVT; (**G**,**H**) non-immune mouse IgG1 control. Scalebars = 50 μm.

Previously, we reported that BCRP is primarily localized to ST in the apical membrane of first trimester floating villi (lacking EVT columns) [10,22]. In this study, we examined BCRP immunolocalization in EVT explant cultures enriched in matrigel where sprouting and migration/invasion of EVTs from the tips of CCTs is identifiable. Immunohistochemistry revealed BCRP immunostaining in ST, CT, CCT, as well as, EVTs invading into the matrigel and at the tips of CCT (Figure 1E,F). Specific trophoblast lineages were histologically identified by comparing BCRP signals in paired serial tissue sections stained with cytokeratin 7 to identify CT, CCT, and ST (Figure 1A,B), and with HLA-G to identify EVTs (Figure 1C,D). Respective IgG controls showed no immunostaining (Figure 1G,H).

### 3.2. ABCG2 Knockdown or Functional Inhibition of BCRP Stimulates EVT Cell Migration (HTR8/SVneo Cells)

**Figure 2:**
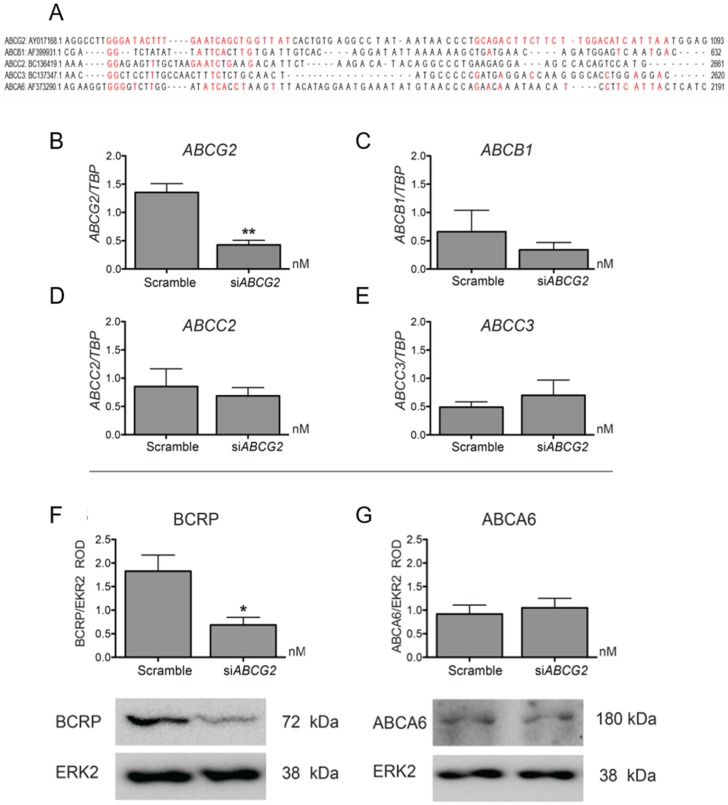
Validation of siRNA mediated knock down of BCRP in HTR8/Svneo cell line. (**A**) Target region of *ABCG2* siRNA sequences (1 & 2 shown in red) aligned with the mRNA of *ABCG2, ABCB1, ABCC2, ABCC3* and *ABCA6* using Multiple Sequence Analysis by Clustal Omega (EMBL-EBI). (**B**–**E**) mRNA analysis post siRNA transfection. *TBP* was used as reference gene for data normalization. *ABCG2* mRNA was significantly downregulated while that of the other transporter genes; *ABCB1*, *ABCC2* and *ABCC3* remained unaffected in siRNA transfected cells compared to the scrambled control ones. (**F**–**G**) Western blot analysis of cells post siRNA transfection. BCRP protein was downregulated 48 h post si*ABCG2* transfection while ABCA6 protein remain unaffected illustrating the specificity of siRNA for BCRP. Graphs show densitometric analysis of BCRP expression (**F**), and ABCA6 (**G**) normalized to total ERK2. Statistical differences were tested by unpaired t test, * *p* < 0.05, ** *p* < 0.01 mRNA n = 4; Protein n = 3 per group.

**Figure 3:**
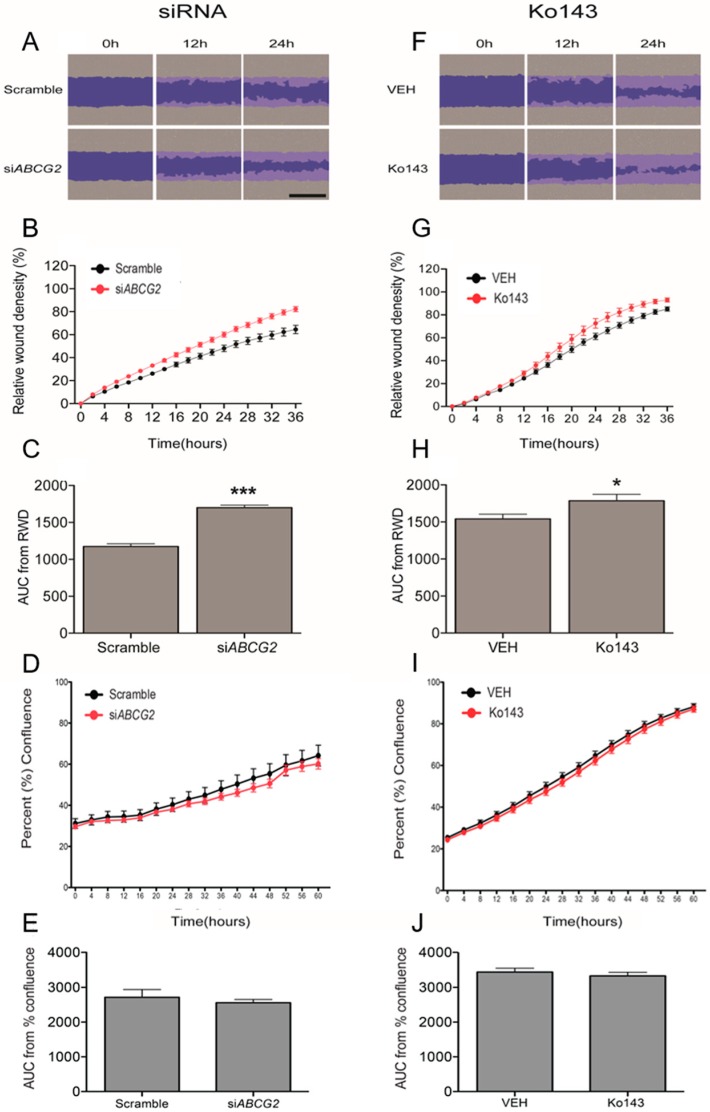
Inhibition of BCRP using siRNA or BCRP specific inhibitor Ko143 increases migration of human EVTs without affecting cell proliferation. HTR8/Svneo cells were cultured in a monolayer in 96-well plates and a wound was made across the diameter of wells using a 96 well Wound Maker. (**A**–**C**) Cells were transfected with 50 nM si*ABCG2* or its scrambled sequence for 48 h prior to wounding and then imaged every 2 h for 36 h to determine relative wound density. (**F**–**H**) Cells were washed, pre-treated with Ko143 or its vehicle (VEH) for 2 h and imaged with IncuCyte™ every 2 h for 36 h to determine relative wound density. (**B**,**G**) Quantification of relative wound density over the period of 36 h. (**C**,**H**) Comparison between treatments is shown by Area under curve analysis of the replicates. Data are expressed as means ± SEM, n = 6 per group. Statistical differences were tested by unpaired *t* test. * *p* < 0.05 versus VEH, *** *p* < 0.001 versus VEH. Scale bars = 600 µm. (**D**,**E**,**I**,**J**) cells were transfected with 50 nM of si*ABCG*2 or scramble control for 24 h and time lapse imaging was performed by IncuCyte^TM^ every 4 h for 60 h. (**D**,**I**) Percent confluence was quantified by incuCyte software and the graph shows mean of n = 6. (**E**,**J**) Comparison between treatments is shown by Area under curve analysis of replicates. Statistical differences were tested by unpaired t-test. No differences were found between the treatments versus the vehicle control.

First, we validated the ability of the si*ABCG2* to reduce the mRNA and protein expression of *ABCG2* and BCRP in HTR8/SVneo cells, which are capable of migration *in vitro*. Significant down-regulation of BCRP (62.37%, Figure 2F) and *ABCG2* (63.85%, Figure 2B) was observed following 48 h of si*ABCG2* duplex treatment. Next, we determined the specificity of siRNA against *ABCG2* by examining the mRNA levels of other related ABC transporters that are known to be expressed in trophoblasts: *ABCB1*, *ABCC2*, and *ABCC3* [29]. The siRNA sequences were with unique specificity against *ABCG2* mRNA. Sequence alignment with other ABC transporters is shown in Figure 2A. We did not find off-target effects of si*ABCG2* at the dose and duration of our experiments (Figure 2C–E). Similarly, we did not find differences in protein expression of another trophoblast-enriched ABC transporter, ABCA6 [29] (Figure 2G), confirming the specificity of the si*ABCG2* procedure. HTR8/SVneo cells were transiently transfected with si*ABCG2* oligos (50 nM) for 48 h (the time point where we observed significant reduction of BCRP protein levels compared to scrambled siRNA control) and were then subjected to wound-scratch assay and IncuCyte^TM^-facilitated imaging and analyses. We found a significant (*p* < 0.001) increase in HTR8/SVneo cell migration in si*ABCG2* transfected cells compared to the scrambled control (Figure 3A–C), with no changes in cell proliferation under similar culture conditions (Figure 3D,E and Supplementary Figure S2), strongly suggesting that BCRP inhibits EVT migration. The inhibition of BCRP function using a BCRP specific inhibitor, Ko143, resulted in a similar significant increase (*p* < 0.05) in trophoblast migration (Figure 3F–H) and did not alter HTR8/SVneo proliferation (Figure 3I,J), confirming that BCRP inhibits EVT migration.

### 3.3. Bacterial and Viral Infection Mimics Activate a Pro-Inflammatory Response in EVTs and Impair ABCG2 and BCRP Expression (HTR8/SVneo Cells)

**Figure 4:**
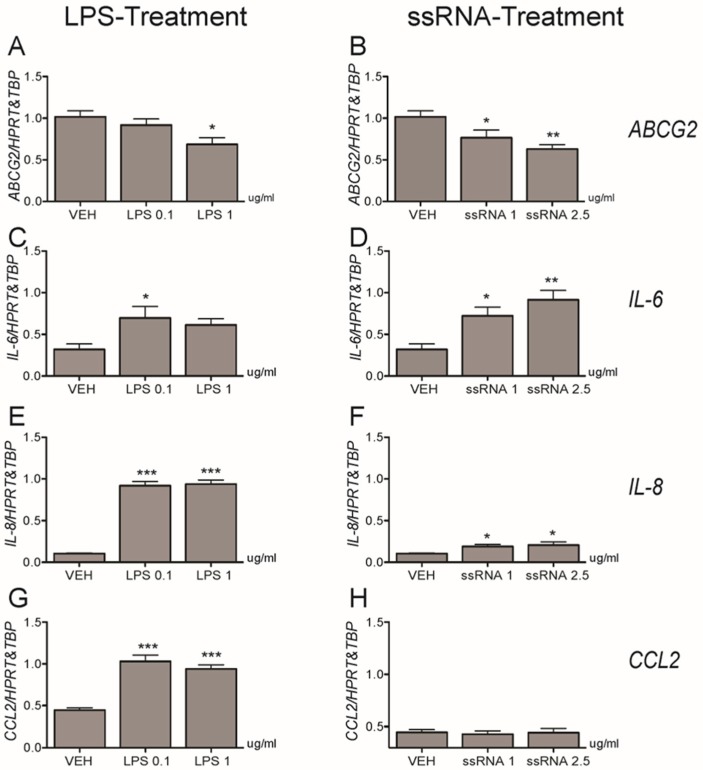
Effects of lipopolysaccharide (LPS, left panel) and single stranded RNA (ssRNA, right panel) on *ABCG2* (**A**,**B**), *IL-6* (**C**,**D**), *IL-8* (**E**,**F**), and *CCL2* (**G**,**H**) expression in human HTR8/SVneo cells. Cells were treated with LPS (0.1 or 1 µg/mL) or ssRNA (1 or 2.5 µg/mL) or their vehicle control (VEH) for 12 h. mRNA expression was assessed by qPCR and the geometric mean of the two reference genes: *HPRT* and *TBP* were used for normalization. Data are expressed as means ± SEM, n = 6 per group. Statistical differences were tested by one-way ANOVA followed by the Newman-Keuls post-hoc test. * *p* < 0.05, ** *p* < 0.01, and *** *p* < 0.001 versus VEH.

**Figure 5:**
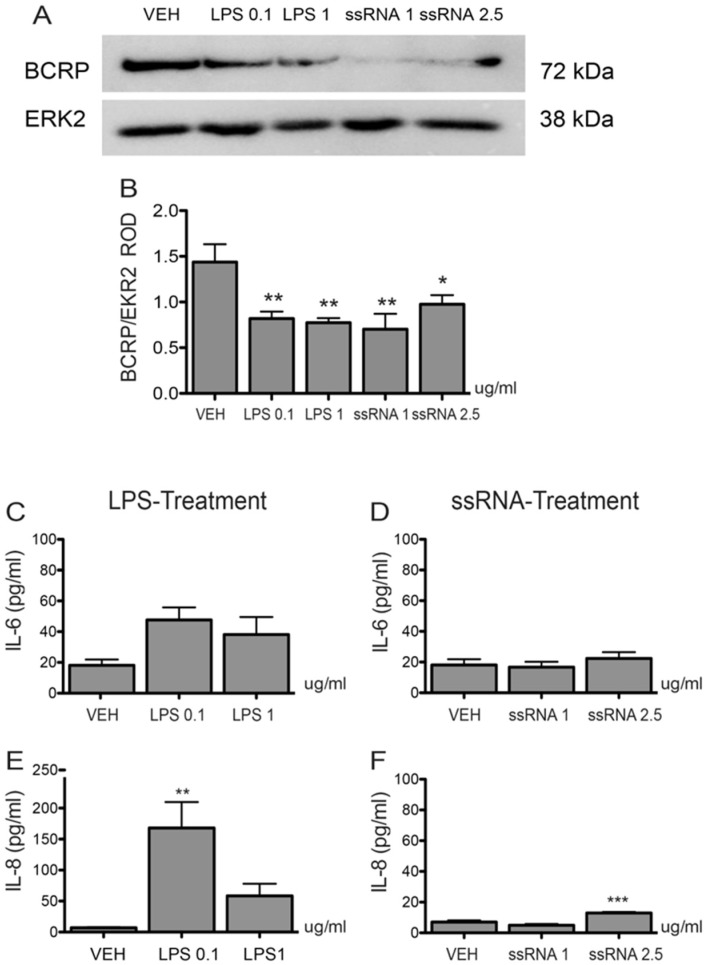
Effect of LPS and ssRNA on BCRP protein expression and IL-6 and IL-8 secretion in HTR8/Svneo cells. Cells were treated with LPS (0.1 or 1 µg/mL) or ssRNA (1 or 2.5 µg/mL) or their vehicle control (VEH) for 12 h. (**A**) Representative western blot for BCRP expression. (**B**) Densitometric analysis of BCRP expression normalized to ERK2. Secreted IL-6 (**C**,**D**) and IL-8 (**E**,**F**) protein levels were measured from HTR8/SVneo cell culture supernatants by enzyme-linked immunosorbent assay (ELISA) 12 h post treatment with 1 µg/mL LPS (**C**,**E**) or 2.5 µg/mL ssRNA (**D**,**F**) or with VEH. Data are expressed as means ± SEM, n = 6 experiments. Statistical differences were tested by one-way ANOVA followed by the Newman-Keuls post-hoc test. * *p* < 0.05, ** *p* < 0.01, *** *p* < 0.001 versus VEH.

LPS treatment (1 µg/mL; 12 h) of HTR8/SVneo cells significantly (*p* < 0.01) decreased *ABCG2* mRNA expression (Figure 4A), while ssRNA exposure repressed *ABCG2* mRNA at both the low and high doses (1 µg/mL: *p* < 0.05, 2.5 µg/mL: *p* < 0.05, Figure 4B), respectively. Since cytokine and chemokine release is the primary systemic response after bacterial or viral infection, we evaluated the LPS and ssRNA inflammatory responses by examining the induction of IL-6, IL-8, and CCL2. Both LPS treatments increased the levels of expression of Interleukin *(IL)-6* mRNA (Figure 4C), *IL-8* (Figure 4E), and C-C Motif Chemokine Ligand 2 (*CCL2*; Figure 4G) mRNA. High dose ssRNA treatment increased levels of *IL-6* (Figure 4D) and *IL-8* mRNA (Figure 4F) at the high dose whereas *CCL2* mRNA expression (Figure 4H) remained unchanged after treatment. Similar to mRNA expression, we found a significant decrease in BCRP protein following 12 h exposure to low and high doses of LPS and ssRNA (Figure 5A,B). There was also a significant increase in IL-8 levels in the culture media after 12 h exposure to low dose LPS (*p* < 0.01) and high dose ssRNA (*p* < 0.001), but no change in IL-6 levels (Figure 5C–F).

### 3.4. LPS and ssRNA Exposure Increases EVT Migration (HTR8/SVneo Cells)

**Figure 6:**
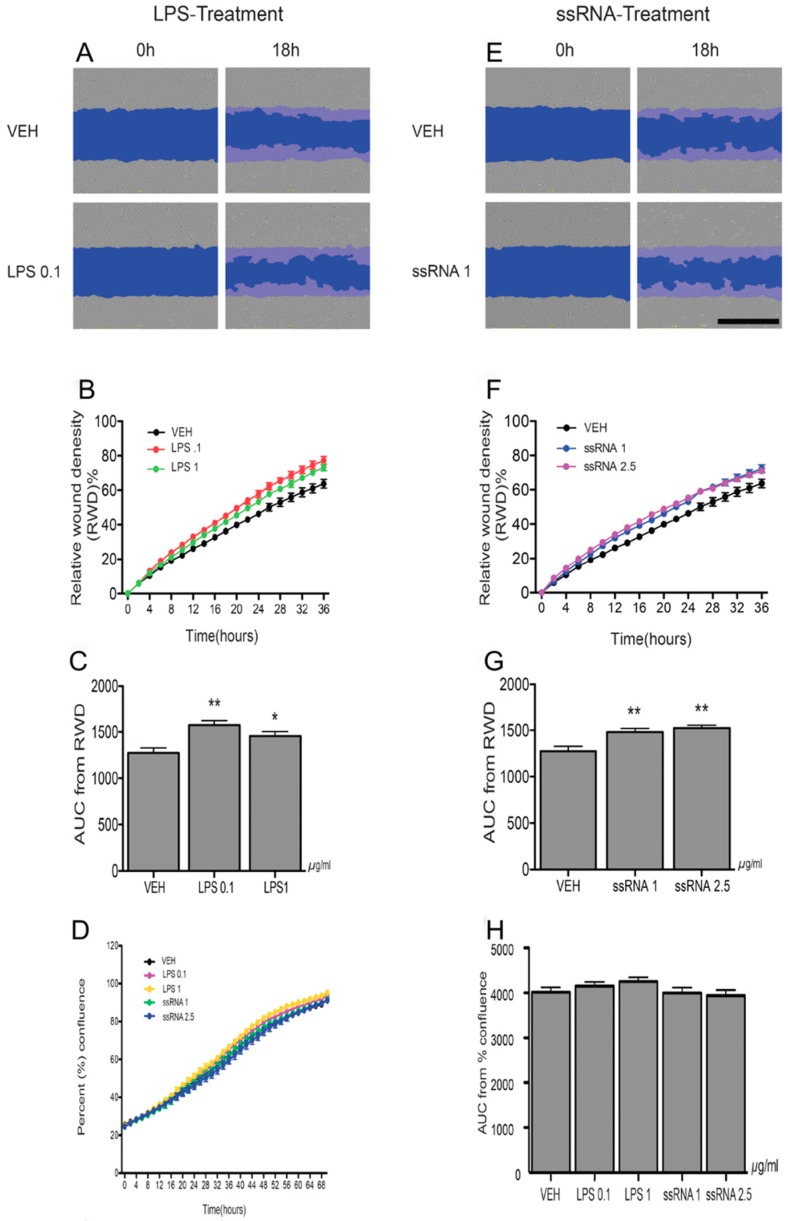
LPS and ssRNA induce human EVT cell migration. HTR8/Svneo cells were cultured in 96-well plates. A wound was made on the cell monolayer using 96-well Wound Maker and cells were imaged with IncuCyte™) every 2 h for 36 h post treatment (see Materials and Methods). (**A**,**E**) Representative images of human EVT migration at 0 h and 16 h. The wound area is artificially coloured blue to highlight the migration of cells into the wound. (**B**,**F**) Graphs show the relative wound density (%) in cells treated with LPS (0.1 or 1 µg/mL) or ssRNA (1 or 2.5 µg/mL) or their vehicle control (VEH) for 24 h. (**C**,**G**) Histograms show comparison between treatments depicted by Area under the curve analysis of the replicates. (**D**,**H**) LPS and ssRNA treatment did not affect EVT cell proliferation. Cells were treated with LPS (0.1 or 1 µg/mL) or ssRNA (1 or 2.5 µg/mL) or their vehicle control (VEH), and time lapse imaging was performed by IncuCyteTM every 2 h for 72 h. (**D**) Percent confluence was quantified by incuCyte software. (**H**) Comparison between treatments is shown by Area under the curve analysis of the replicates. Data are expressed as means ± SEM, n = 6 per treatment group. Statistical differences were tested by one-way ANOVA followed by the Newman-Keuls post-hoc test. * *p* < 0.05, ** *p* < 0.01 versus VEH. Scale bars = 600 µm.

Since LPS and ssRNA decreased BCRP expression in EVT-like cells, we undertook a series of experiments to determine if LPS and ssRNA treatments affect EVT migration. The wound-scratch assay revealed that cells exposed to low or high doses of LPS (Figure 6A–C) and ssRNA (Figure 6E–G) exhibited a significantly higher % wound density than cells treated with the vehicle. Analysis of data by the area under the curve (AUC) showed significant (*p* < 0.01) increases in the migration of cells exposed to LPS (Figure 6C) or ssRNA (Figure 6G), with no changes in cell proliferation (Figure 6D,H and Supplementary Figure S2).

### 3.5. ABCG2 Knockdown Stimulates EVT Cell Migration in Placental Explants

**Figure 7:**
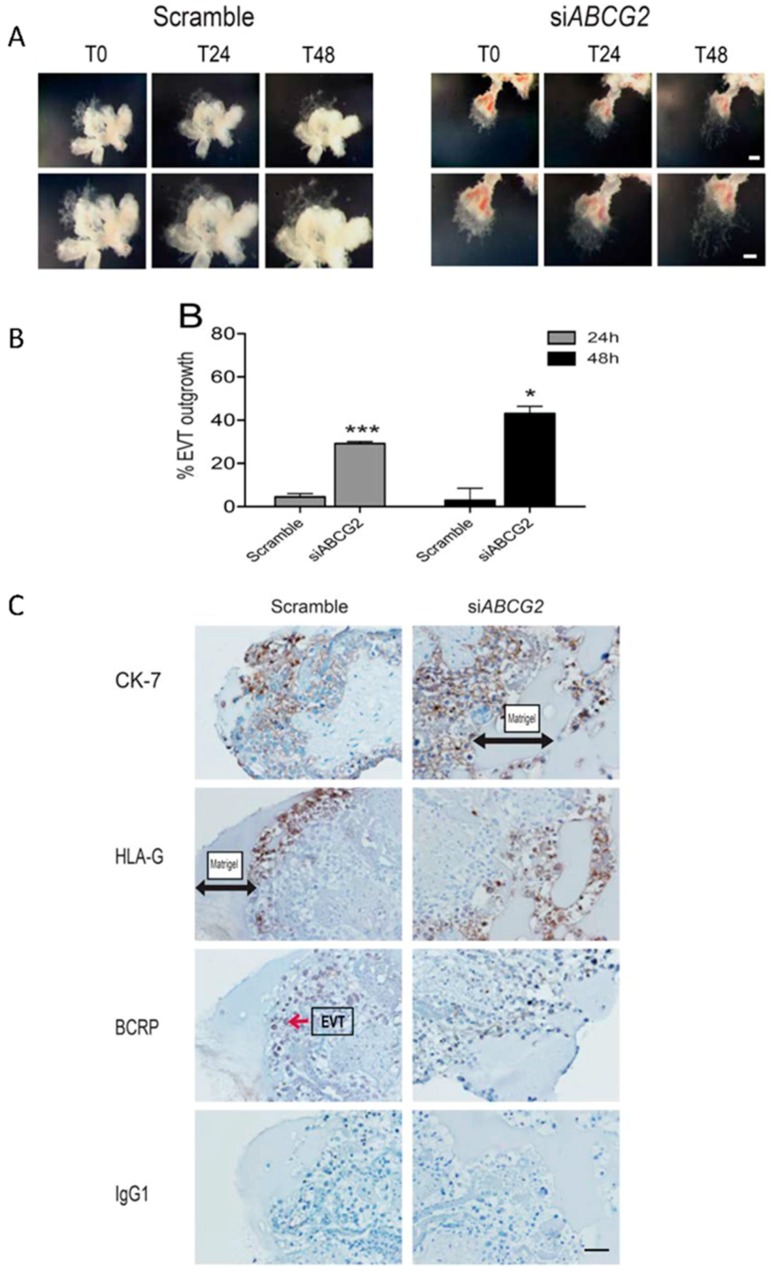
*ABCG2* knockdown stimulated EVT cell migration in human placental explants. (**A**) Representative images of 7-week placental explants treated with Scrambled (control) siRNA or *siABCG2* at T0, T24, and T48. (**B**) Graph shows mean ± SEM (n = 3 women). Statistical significance between scramble control and *siABCG2* was determined by paired *t*-test, * *p* < 0.05 and *** *p* < 0.001. Scale bar = 200 µm. (**C**) Representative immunohistochemistry images from tissue sections of first trimester human placental explants cultured on Matrigel and treated with 50 nM of control siRNA or *siABCG2* for 48 h (n = 3), showing downregulation of BCRP in the *siABCG2* tissue versus the control. Cytokeratin (CK-7) staining is shown as a marker of trophoblasts (including CCT, CT, STB, and EVT), and HLA-G as a marker of EVTs. Scale bars = 50 µm.

We used human placental explants as an *ex-vivo* model of human placenta to determine the physiological significance of BCRP on EVT function and placentation. Following the transfer of first trimester human placental explants to Matrigel coated transwell inserts, villi with visible signs of EVT outgrowths were treated with *siABCG2* or control oligos and were assessed at the time of transfection (T0), at 24 h (T24), and 48 h (T48) post-transfection. EVT migration was determined by comparing the percent EVT outgrowth in the control versus *siABCG2* treated explants. There was a substantial reduction of BCRP expression in the placental villi of *siABCG2* treated explants compared to the controls, as determined by immunohistochemistry (Figure 7). Silencing of *ABCG2* resulted in significantly greater expansion (migration and invasion) of the EVT outgrowth on the Matrigel at 24 h (*p* < 0.001) and 48 h (*p* < 0.05) compared to the control (Figure 7A,B). HLA-G was used as a marker of EVT to verify the migration and invasion of EVTs across the Matrigel in *siABCG2* versus control explants, and CK7 was used as a trophoblast marker (Figure 7C).

## 4. Discussion

Our study provides new insights into the regulation of EVT migration during early placental development. We report, for the first time, that BCRP, which is well known for its role as an efflux transporter, inhibits EVT cell migration in HTR8/SVneo (EVT-like) cells and in first trimester derived EVTs. In addition to being present in STs, BCRP is also expressed in CCTs and EVTs in human placenta during the first trimester, implicating that it has a role in EVT function. Inhibition of BCRP with si*ABCG2* or Ko143 increased cell migration in the HTR8/SVneo (EVT-like) cell line over 36 h, without affecting proliferation. We also demonstrated that LPS and ssRNA exposure decreased *ABCG2* and BCRP expression in EVTs and stimulated EVT migration. Together, these data suggest that BCRP functions as an inhibitor of EVT migration and that infection may lead to increased EVT migration.

In the present study, we effectively knocked down BCRP expression using siRNA duplex technology, which resulted in increased migration of EVT cells, with no alteration in the proliferation index. It is important to note that we used a novel automated approach to monitor and analyze the migration of EVT cells. We calculated the % wound density and the area under the curve generated from the data recorded every 2 h over 36 h using IncuCyte™ technology [15]. Pharmacological inhibition of BCRP resulted in a similar outcome. Interestingly, this contrasts with reports from cancer cell lines, which suggest that BCRP increases the migration of glioma and pancreatic cancer cells [16,17,30] and inhibits the proliferation in other epithelial-like cancer cell lines [14]. These differences may result from the fact that the cancer cell lines are derived from human tumors, whereas HTR8/SVneo cells are derived from physiological EVTs [23], thus illustrating different actions of BCRP modulating cell migration in physiological and in neoplastic phenotypes. Further, diverse cancer cells may commonly exhibit increased basal expression of *ABCG2* and BCRP, an important mechanism conferring multidrug resistance to chemotherapeutic drugs [31]. This is postulated to be an adaptation to the rearrangement of intracellular signaling pathways undertaken by malignant cells to promote invasiveness [32].

In a previous study, we reported that P-glycoprotein (P-gp), another ABC transporter related to ST-mediated fetal protection, reduces HTR8/SVneo cell proliferation and invasion [20]. While the contrasting actions of BCRP and P-gp in early placentation require further investigation, it is attractive to speculate that P-gp drives placentation by enhancing EVT migration/invasion, while BCRP likely controls the extent of EVT penetration within the decidua. The mechanisms by which BCRP modulates EVT migration remains to be determined. BCRP may regulate the transport and activity of substrates which are critical to cell migration (such as extracellular matrix (ECM) adhesion molecules). P-gp and BCRP are known to interact with CD44 to modulate cancer cell migration/invasion [33,34]. It is also possible that P-gp and BCRP have shared mediators controlling EVT cell migration. For example, CD44 and hyaluronic acid (HA) mediate cell–cell/cell–matrix adhesion and, hence, affect cell migration [35,36,37], and it is documented that CD44/HA on the cell surface of EVTs are important mediators of EVT invasion [38]. The STRING database of protein–protein interaction indicates interaction between CD44 and BCRP (https://string-db.org/cgi/network.pl?taskId=okGQMLlRXDFw). Based on this, it is possible to speculate that BCRP competes with P-gp for CD44 binding, interferes with the CD44/HA mediated adhesion properties of EVTs, and, hence, impedes EVT migration/invasion. This possibility requires further investigation.

In the present study, LPS treatment, which models bacterial infection via TLR-4 activation [39], decreased *ABCG2* mRNA and BCRP protein expression in HTR-8/SVneo cells. This indicates that bacterial infection in early human pregnancy has the potential to affect EVT migration through the modulation of BCRP expression. This pattern of BCRP inhibition is consistent with our previous report showing that *ABCG2* and BCRP expression was decreased 24 h after exposure of first trimester human explants to LPS [12]. In the rat, previous studies have shown LPS to down-regulate placental *Abcg2* mRNA [40]. No studies have investigated the effect of LPS on BCRP activity, however in mid-gestation, LPS inhibited the P-gp (encoded by *Abcb1a* and *Abcb1b* in rodents) efflux transporter in the mouse placenta [41]. Together, these studies suggest that the regulation of placental *ABCG2* by LPS is gestational age-dependent. Importantly, it also indicates that bacterial infection has the potential to compromise normal EVT migration and fetal protection by inhibiting BCRP expression in EVTs and in the ST, respectively.

Exposure to ssRNA, which models viral infections and induces trophoblast pro-inflammatory responses via the activation of TLR8 and non-TLR8 signaling pathways [25], also decreased *ABCG2* mRNA and BCRP protein expression in EVTs. This indicates that viral infection may, via decreased expression of BCRP, disrupt normal EVT migration. There are no previous reports of ssRNA modulating placental BCRP expression, though Poly (I:C), a synthetic analog of double stranded RNA (dsRNA) that acts via TLR-3 activation [42], has been shown to decrease *ABCG2* in third trimester human placental explants [12] and in the late gestation rat placenta [40]. No studies have assessed the effect of Poly (I:C) on BCRP function. However, Poly (I:C) inhibited P-gp activity in the fetal mouse blood–brain barrier, but did not alter placental P-gp function [43], suggesting that the effects of dsRNA viral infection on P-gp mediated fetal protection are tissue barrier dependent. Future studies investigating the role of ssRNA regulating P-gp and BCRP function in developing barriers are warranted.

Maternal infection with single-stranded RNA viruses capable of crossing the placental barrier, i.e., zika (ZIKV) and rubella viruses [44,45], elicit severe obstetric outcomes including microencephaly, cerebral palsy, intellectual disability, and other sequelae [46,47]. In the present study, we showed that infection with ssRNA (which also include influenza (flu virus), rhinovirus (common cold virus), retrovirus (HIV and HTLV1), and coronavirus (upper-respiratory illnesses virus)) (Potter et al. 2015; Luo et al. 2018) in early-pregnancy has the potential to disrupt normal EVT migration and ultimately placentation, placental function, and fetal development. In this context, human placentae infected by ZIKV exhibit marked villous immaturity and increased the ratio of syncytial sprouts (syncytial nuclei aggregation at the outer trophoblastic wall) [48,49]. This provides pathological evidence of uteroplacental malperfusion, typically associated with deranged EVT migration into the decidualized endometrium, resulting in poor myometrial spiral arteries remodeling and reperfusion [50]. Further, viral, bacterial, and parasitic infections have been related to the enhancement of the likelihood of PE, through the modulation of pro-inflammatory cytokines, oxidative stress, and anti-angiogenic proteins at the early maternal fetal interface [51]. 

We have shown that ssRNA increased HTR-8/SVneo migration. Importantly, no previous studies have investigated the impact of ssRNA on trophoblast (EVT) migration or the migration of other normal somatic cells. However, studies on cancer cells have shown that ssRNA does not affect the migration and invasion of melanoma cells (Liu et al., 2018).

In the present study, we provide evidence that *ABCG2* and BCRP inhibit EVT migration and are repressed by bacterial and viral infective challenges. Our results suggest that the downregulation of BCRP may be involved in the cellular pathways by which infection increases EVT cell migration. However, we cannot rule out that other ABC transporters, modulated by LPS and ssRNA in our EVT culture systems, may have contributed to the increased migration rates herein observed, following infective challenges. Further, *ABCG2* and BCRP are highly responsive to a number of cytokines [9] and, in the present study, we have shown that LPS and ssRNA increased IL-6 (mRNA level only) and IL-8 secretion (mRNA and protein), suggesting that these cytokines inhibit or have additional inhibitory effects on *ABCG2* and BCRP expression in EVTs, a hypothesis that requires further investigation.

**Figure 8:**
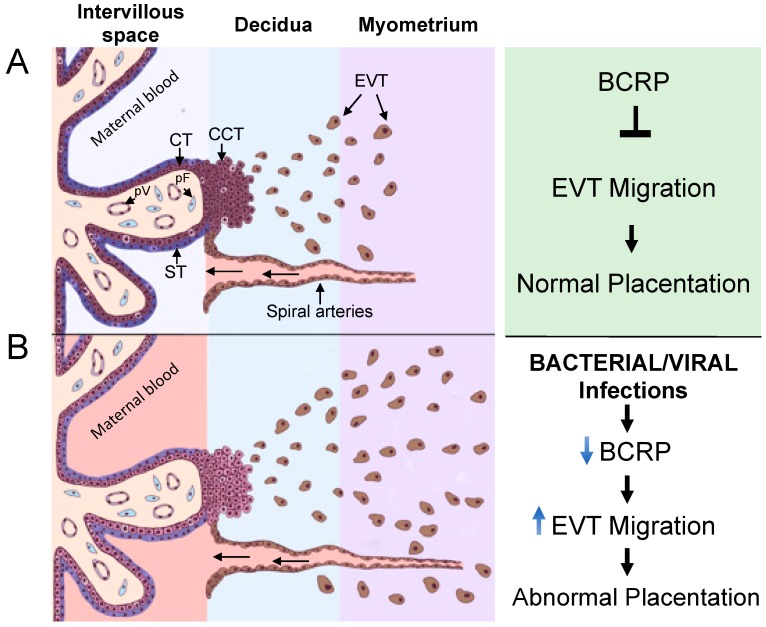
Hypothetical model depicting the impact of bacterial/viral infection mediated downregulation of BCRP on human placentation (**A**) BCRP decrease cell migration and when disrupted by bacterial and viral infections, (**B**) may increase EVT migration and lead to abnormal placentation.

In conclusion, we have identified a novel role for BCRP in early placentation (Figure 8). The present study has demonstrated that EVTs express *ABCG2* and BCRP and that BCRP limits EVT migration. Bacterial and viral infection results in a decrease in *ABCG2* and BCRP in the EVT and a concomitant increase in EVT migration, suggesting that BCRP plays a role in infection-related alterations in EVT function. Such mechanisms may underlie the etiology of pregnancy complications.

## Figures and Tables

**Table 1:** List of primers used in this study.

Primer Name	Sequence	Reference
*ABCG2*	Forward: 5′-TGGAATCCAGAACAGAGCTGGGGT-3′	Lye et al., 2018
	Reverse: 5′-AGAGTTCCACGGCTGAAACACTGC-3′	
*IL-6*	Forward: 5′-TGCAGAAAAAGGCAAAGAAT-3′	Potter et al., 2015
	Reverse: 5′-CTGACCAGAAGAAGGAATGC-3′	
*IL-8*	Forward: 5′-TGGGAACAAGAGGGCATCTG-3′	Lye et al., 2015
	Reverse: 5′-CCACCACTGCATCAAATTCATG-3′	
*CCL2*	Forward: 5′-TTCATTCCCCAAGGGCTCGCTCA-3′	Lye et al., 2015
	Reverse: 5′-AGCACAGATCTCCTTGGCCACAA-3′	
*TLR-4*	Forward: 5′-ATTTGTCTCCACAGCCACCA-3′	Lye et al., 2015
	Reverse: 5′-ACAGGAAACCCCATCCAGAG-3′	
*HPRT*	Forward: 5′-TGA CAC TGG CAA AAC AATGCA-3′	Drewlo et al., 2012
	Reverse: 5′-GGT CCT TTTCAC CAG CAA GCT-3′	
*TBP*	Forward: 5′-TGC ACA GGA GCC AAG AGT GAA-3′	Drewlo et al., 2012
	Reverse: 5′-CAC ATC ACA GCT CCC CAC CA-3′	

**Table 2:** List of *ABCG2*-siRNAs used in this study.

Number		Sequence
1	Antisense	AUAACCAGCUGAUUCAAAGUAUCCC
	Sense	GGGAUACUUUGAAUCAGC UGGUUAU
2	Antisense	UAAUGAUGUCCAAGAAGAAGUCUGC
	Sense	CAGACUUCUUCUUGGACAUCAUUA
3	Antisense	GGAGGCAAAUCUUCGUUAUTT
	Sense	AUAACGAAGAUUUGCCUCCTT
Negative Control	Antisense	GCGACGAUCUGCCUAAGAUdTdT
	Sense	AUCUUAGGCAGAUCGUCGCdTdT

Provided by Shanghai GenePharma, China.

## Supplementary Materials

The following are available online at https://www.mdpi.com/2073-4409/8/10/1150/s1, Figure S1: Representative Images of cell proliferation assay following si*ABCG2*, scrambled siRNA, Ko143 or VEH exposure at 0 and 24 h N = 6, Scale bars = 400 µm. Figure S2: Representative Images of cell proliferation assay following LPS or ssRNA or VEH exposure at 0 and 24 h. N = 6, Scale bars = 400 µm.
