# Supplementary figures and images for: Breast Cancer Resistance Protein (BCRP/ABCG2) Inhibits Extra Villous Trophoblast Migration: The Impact of Bacterial and Viral Infection
Source: Cells. 2019 Sep 26;8(10):1150. doi: 10.3390/cells8101150 (PMC6829363; doi:10.3390/cells8101150)

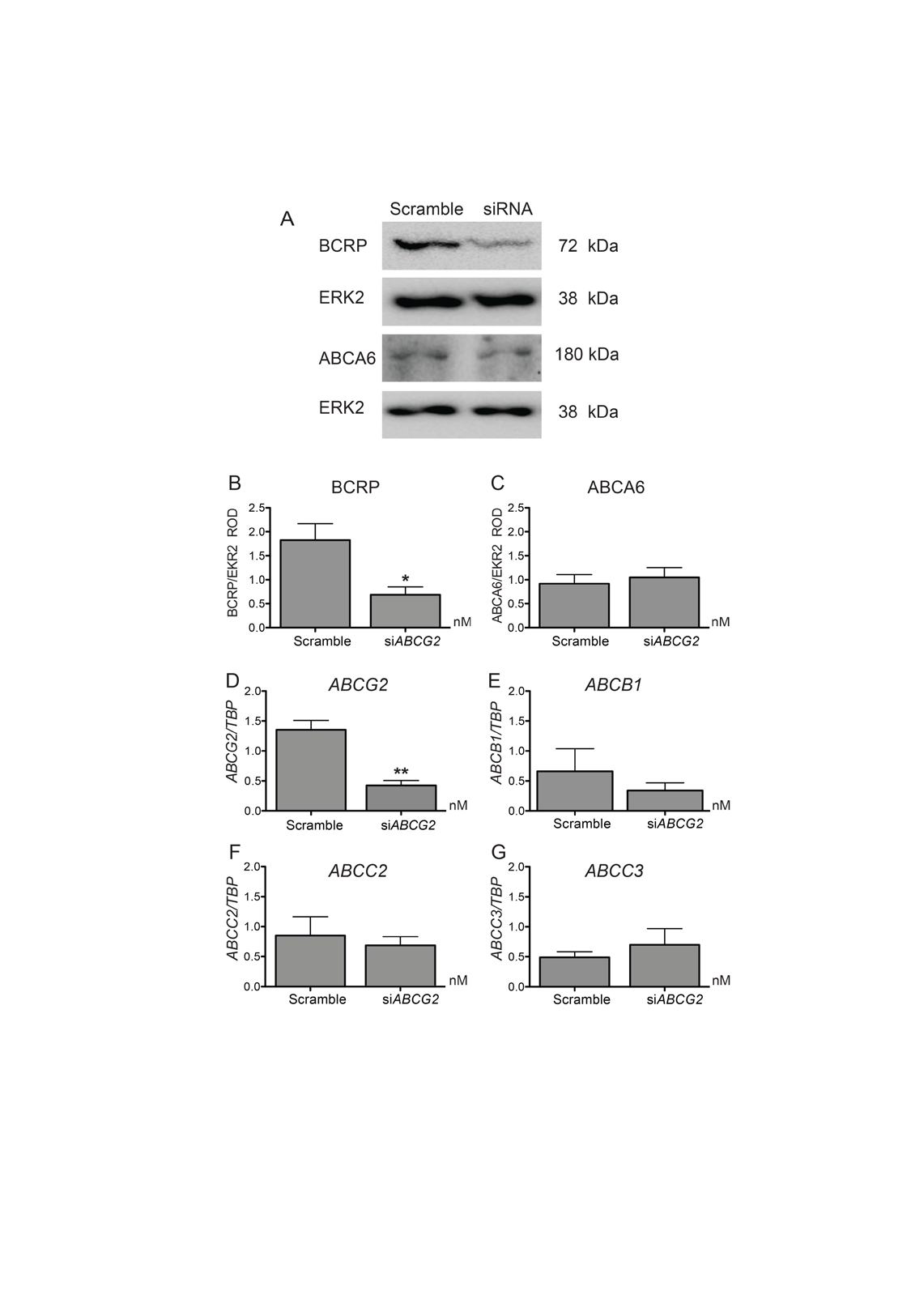

Supplement: Supplementary file 1 [file cells-08-01150-s001.zip › Supplementary Figures Combined.tiff]
